# Comparison of efficacy and safety of first-line palliative chemotherapy with EOX and mDCF regimens in patients with locally advanced inoperable or metastatic HER2-negative gastric or gastroesophageal junction adenocarcinoma: a randomized phase 3 trial

**DOI:** 10.1007/s12032-015-0687-7

**Published:** 2015-09-09

**Authors:** Sebastian Ochenduszko, Miroslawa Puskulluoglu, Kamil Konopka, Kamil Fijorek, Katarzyna Urbanczyk, Andrzej Budzynski, Maciej Matlok, Agata Lazar, Anna Sinczak-Kuta, Michal Pedziwiatr, Krzysztof Krzemieniecki

**Affiliations:** Department of Oncology, Jagiellonian University Medical College, Ul.Sniadeckich 10, 31-531 Kraków, Poland; Department of Clinical Oncology, University Hospital in Krakow, Kraków, Poland; Department of Statistics, Cracow University of Economics, Kraków, Poland; Department of Pathology, Jagiellonian University Medical College, Kraków, Poland; 2nd Department of General Surgery, Jagiellonian University Medical College, Kraków, Poland

**Keywords:** Gastric cancer, Palliative chemotherapy, EOX, mDCF, Toxicity, Safety

## Abstract

The aim of the study was to compare efficacy and safety of first-line palliative chemotherapy with (EOX) epirubicin/oxaliplatin/capecitabine and (mDCF) docetaxel/cisplatin/5FU/leucovorin regimens for untreated advanced HER2-negative gastric or gastroesophageal junction adenocarcinoma. Fifty-six patients were randomly assigned to mDCF (docetaxel 40 mg/m^2^ day 1, leucovorin 400 mg/m^2^ day 1, 5FU 400 mg/m^2^ bolus day 1, 5FU 1000 mg/m^2^/d days 1 and 2, cisplatin 40 mg/m^2^ day 3) or EOX (epirubicin 50 mg/m^2^ day 1, oxaliplatin 130 mg/m^2^ day 1, capecitabine 1250 mg/m^2^/d days 1–21). The primary endpoint was overall survival. The median overall survival was 9.5 months with EOX and 11.9 months with mDCF (*p* = 0.135), while median progression-free survival was 6.4 and 6.8 months, respectively (*p* = 0.440). Two-year survival rate was 22.2 % with mDCF compared to 5.2 % with EOX. Patients in the EOX arm had more frequent reductions in chemotherapy doses (34.5 vs. 3.7 %; *p* = 0.010) and delays in subsequent chemotherapy cycles (82.8 vs. 63.0 %; *p* = 0.171). There was no statistically significant difference in the rates of grade 3–4 adverse events (EOX 79.3 vs. mDCF 61.5 %; *p* = 0.234). As compared with the mDCF, the EOX regimen was associated with more frequent nausea (34.5 vs. 15.4 %), thromboembolic events (13.8 vs. 7.7 %), abdominal pain (13.8 vs. 7.7 %) and grades 3–4 neutropenia (72.4 vs. 50.0 %), but lower incidences of anemia (44.8 vs. 61.5 %), mucositis (6.9 vs. 15.4 %) and peripheral neuropathy (6.9 vs. 15.4 %). In conclusion, the mDCF regimen was associated with a statistically nonsignificant 2.4-month longer median overall survival without an increase in toxicity. This trial is registered at ClinicalTrials.gov, number NCT02445209.

## Introduction

Worldwide, gastric cancer is the fourth most common malignancy and the second leading cause of cancer death [1]. Around 2/3 of cases are diagnosed in locally advanced or metastatic stage, where palliative chemotherapy is the main treatment option. The prognosis for patients with advanced gastric cancer receiving no treatment is poor, with a median overall survival of 3–5 months [2–4]. A few randomized trials and meta-analysis showed an improvement in weighted average survival of about 6 months in patients treated with palliative chemotherapy [2–5], their prognosis, however, is still poor with 5-year survival rates of 5–20 % and median overall survival <12 months. There is no single, global standard regimen for the first-line treatment of advanced disease. The most common chemotherapy combinations in the first-line setting consist of two or three drugs and are cisplatin and fluoropyrimidine based. The meta-analysis showed an improvement in weighted average survival of approximately 2 months with addition of antracycline to cisplatin and 5FU regimen [5], and ECF combination (epirubicin/cisplatin/5FU) became a standard in many countries for treating this disease. In a randomized phase 3 trial, oxaliplatin and capecitabine were non-inferior to cisplatin and 5FU, respectively, and the EOX regimen (epirubicin/oxaliplatin/capecitabine) was associated with the longest overall survival of 11.2 months [6]. The addition of docetaxel to cisplatin and 5FU (DCF regimen) improved survival of patients compared to cisplatin and 5FU alone in a phase 3 trial [7]; however, this three-drug combination was associated with a significant toxicity. The modified DCF (mDCF) regimen has recently been shown to have at least equal efficacy and lower toxicity compared to standard DCF chemotherapy in a phase 2 trial [8]. The EOX regimen is usually administered in the chemotherapy day unit with epirubicin and oxaliplatin given intravenously and capecitabine administered in the ambulatory setting, and is repeated every 3 weeks. Thus, the regimen seems appropriate for patients who wish to maintain high life activity without being hospitalized. On the other hand, the mDCF regimen, repeated every 2 weeks, usually requires at least a 3-day hospitalization due to continuous intravenous administration of 5FU and hydration for cisplatin. The aim of the study was to compare the efficacy and toxicity of the first-line palliative three-drug chemotherapy with EOX and mDCF regimens, respectively, in patients with (HER2) human epidermal growth factor receptor 2 negative, locally advanced inoperable or metastatic gastric or gastroesophageal junction adenocarcinoma.

## Patients and methods

### Patient characteristics

This was a randomized, single-centre phase 3 study. Patients older than 18 years of age were eligible for inclusion if they had histologically confirmed inoperable locally advanced, recurrent or metastatic adenocarcinoma of the stomach or gastro-oesophageal junction; (ECOG) Eastern Cooperative Oncology Group performance status 0–2; adequate renal, hepatic and hematologic function; and measurable or non-measurable disease according to the Response Evaluation Criteria in Solid Tumors (RECIST). Patients with intraoperatively confirmed intraperitoneal metastases but without detectable disease in radiological studies were also eligible. Major exclusion criteria included: HER2-positive tumors, previous chemotherapy for metastatic or locally advanced disease, congestive heart failure, significant dysphagia that would preclude oral administration of capecitabine, concurrent cancer and evidence of brain metastases. Tumors were tested for HER2 status with immunohistochemistry (IHC) and fluorescence in situ hybridisation (FISH). Patients with IHC3 + or IHC2 + and FISH-positive results were excluded from the study. The protocol of the study was approved by a university ethics committee. Patients provided written informed consent, and the study was carried out in accordance with Good Clinical Practice guidelines and the provisions of the Declaration of Helsinki. All the data were collected and managed by the physicians of the Department of Oncology at the University Hospital in Krakow. This was an academic study with no external sponsors.

### Treatment

With the use of random permuted blocks, patients who fulfilled all eligibility criteria were assigned (1:1) to either EOX or mDCF chemotherapy. The EOX regimen was given every 3 weeks, initially for a maximum of eight cycles (24 weeks of treatment). It consisted of epirubicin 50 mg/m^2^ (intravenous bolus), followed by oxaliplatin 130 mg/m^2^ (2-h intravenous infusion); capecitabine was administered orally, twice daily at the dose of 625 mg/m^2^ for 21 days. The mDCF regimen was administered every 2 weeks, initially for a maximum of 12 cycles (24 weeks of treatment), docetaxel 40 mg/m^2^ (intravenous infusion over 60 min) on day 1, followed by leucovorin 400 mg/m^2^ (intravenous infusion over 120 min) on day 1, followed by 5-fluorouracil 400 mg/m^2^ (intravenous bolus) on day 1, and then 5-fluorouracil 1000 mg/m^2^/day continuous intravenous infusion on day 1 and day 2, followed by cisplatin 40 mg/m^2^ (intravenous infusion over 60 min) on day 3. All patients received appropriate hydration and premedication which were at the discretion of the treating physician. Chemotherapy dose adjustments and treatment delays were allowed and were at the discretion of the treating physician. Treatment continued until disease progression, unacceptable toxicity, death or consent withdrawal. Patients who experienced a long-term response to the initial eight cycles of EOX or 12 cycles of mDCF chemotherapy had the possibility of being rechallenged with the same regimen (the decision was at the discretion of a treating physician). After progression, eligible patients were treated with the second-line irinotecan monotherapy.

### Evaluation and outcomes

Before random assignment, a complete evaluation was carried out; it included full medical history, physical examination, complete blood count, serum biochemical analysis and electrocardiography. Echocardiography was obligatory in patients with signs or history of heart failure or history of coronary artery disease. Baseline tumor assessments, including computed tomography of the abdomen (and pelvis in female patients) and chest X-ray, were performed within 28 days before treatment initiation. If the chest X-ray was suspicious for metastases or the patient presented symptoms of metastases in the chest, a computed tomography of the chest was performed. Tumor assessments were initially planned to be repeated every 8–9 weeks during the active treatment phase of the study. However, taking into account it was not a sponsored trial and access to CT scans was limited, we adopted our routine clinical strategy to perform CT scans every 8–12 weeks. Disease progression could also be evaluated based on clinical symptoms and urgent CT was requested whenever needed. After the active treatment phase of the study, subsequent CT scans were performed every 12 weeks (±2 weeks) or whenever needed depending on the symptoms. However, progression-free survival was not a primary endpoint of the study. Toxicities were graded according to the Common Toxicity Criteria Adverse Events (CTCAE) version 4.0.

The primary endpoint was overall survival defined as time from randomization until death from any cause. Secondary endpoints were progression-free survival and safety. All randomized patients received study medication at least once and were included in the analysis. Patients without an event (death) were censored at the date that they were last known to be alive.

### Statistical analysis

Continuous variables were presented as means and standard deviations. Categorical variables were presented as counts and percentages. For continuous variables, statistical significance of differences between two independent groups was assessed using *t* test. For two categorical variables, the Fisher exact test was used. The Kaplan–Meier method was used to estimate the survival distributions. Survival distributions were compared using the log-rank test. A *p* value <0.05 was considered an indication of a statistically significant result. No adjustment for multiple comparisons was made. All statistical analyses were performed using R 3.0

## Results

### Patients

**Fig. 1:**
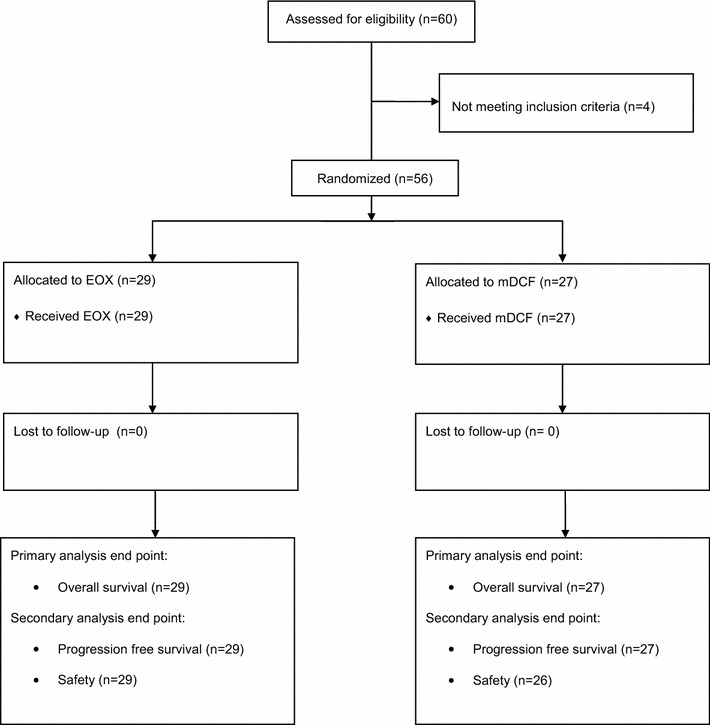
CONSORT diagram depicting the trajectory of the trial

Between September 2010 and February 2014, fifty-six patients (29 in the EOX arm and 27 in the mDCF arm) were randomly assigned and received at least one cycle of the treatment (Fig. 1). Both treatment groups were well balanced for baseline characteristics (Table 1), except for malnutrition as assessed by initial body mass index (BMI) (mild malnutrition according to BMI 3.5 % in EOX arm vs. 18.5 % in mDCF arm; *p* = 0.064) and lymphocytes level at study entry <1500/μL (31.0 vs. 48.1 % in EOX and mDCF arm, respectively; *p* = 0.300); however, these differences were not statistically significant. Most patients had metastatic disease and more than 50 % of patients in each arm have undergone gastrectomy (primary tumor resection) as part of curative or palliative treatment. Significantly more patients in the mDCF arm presented with metastases in the liver (48.1 vs. 17.2 %; *p* = 0.029).

**Table 1:** Patient demographics and clinical characteristics

	EOX N = 29	mDCF N = 27	*p* value
Mean age (years), (SD)	57.9 (10.8)	60.3 (9.11)	0.365
Men	16 (55.2 %)	13 (48.1 %)	0.796
ECOG performance status			1.000
0–1	26 (89.7 %)	25 (92.6 %)	
2	3 (10.3 %)	2 (7.4 %)	
Mean BMI at study entry, (SD)	23.5 (3.80)	23.6 (4.64)	0.893
BMI groups at study entry			0.064
17–18.99 (mild malnutrition)	1 (3.5 %)	5 (18.5 %)	
19–24.99 (normal)	20 (69.0 %)	11 (40.7 %)	
≥25.0 (overweight)	8 (27.6 %)	11 (40.7 %)	
Lymphocytes at study entry			0.300
<1500 (malnutrition)	9 (31.0 %)	13 (48.1 %)	
≥1500 (normal)	20 (69.0 %)	14 (51.9 %)	
Previous curative treatment of gastric/GEJ adenocarcinoma	2 (6.9 %)	1 (3.7 %)	1.000
Gastrectomy	16 (55.2 %)	14 (51.9 %)	0.898
Extent of disease at study entry			0.343
Locally advanced	1 (3.4 %)	3 (11.1 %)	
Metastatic	28 (96.6 %)	24 (88.9 %)	
Location of metastases			
Distant lymph nodes	13 (44.8 %)	13 (48.1 %)	1.000
Liver	5 (17.2 %)	13 (48.1 %)	0.029
Lungs	1 (3.4 %)	2 (7.4 %)	0.605
Peritoneum	16 (55.2 %)	12 (44.4 %)	0.593
Ovaries	2 (6.9 %)	2 (7.4 %)	1.000
Pleura	2 (6.9 %)	1 (3.7 %)	1.000
Other	3 (10.3 %)	1 (3.7 %)	0.612
Number of metastatic sites involved			0.688
0 or 1	16 (55.2 %)	13 (48.1 %)	
2	11 (37.9 %)	10 (37.0 %)	
≥3	2 (6.9 %)	4 (14.8 %)	
Lauren classification			0.765
Intestinal	5 (17.2 %)	6 (22.2 %)	
Diffuse	10 (34.5 %)	10 (37.0 %)	
Mixed	5 (17.2 %)	6 (22.2 %)	
Unknown	9 (31.0 %)	5 (18.5 %)	

### Chemotherapy

The mean duration of the first-line chemotherapy (EOX or mDCF) did not differ between the groups (5.42 months for EOX vs. 4.56 months for mDCF; *p* = 0.237). Dose reductions due to toxicity occurred in ten patients (34.5 %) with EOX and only one patient (3.7 %) with mDCF (*p* = 0.010). Treatment delays were also more frequent in the EOX arm (82.8 vs. 63.0 %; *p* = 0.171), but this difference did not reach statistical significance. The most common adverse events leading to dose reductions and treatment delays were neutropenia, thrombocytopenia and fatigue. The second-line treatment with irinotecan monotherapy was administered to 15 patients (51.7 %) in the EOX arm compared to 11 patients (40.7 %) in the mDCF arm (*p* = 0.436). Two patients (one patient in each arm) received third-line palliative chemotherapy (Table 2).

**Table 2:** Analysis of efficacy

	EOX	mDCF	*p* value
Median overall survival, months (95 % CI)	9.5 (8.3–13.6)	11.9 (10.4–14.8)	0.135^a^
1-year survival rate, % (95 % CI)	31.0 (18.0–53.4)	44.4 (29.2–67.8)	
2-year survival rate, % (95 % CI)	5.2 (0.8–32.6)	22.2 (11.0–45.0)	
Median progression-free survival, months (95 % CI)	6.4 (5.3–9.0)	6.8 (3.3–9.5)	0.440^a^
Mean duration of first-line chemotherapy, months (SD)	5.42 (1.85)	4.56 (3.28)	0.237
At least one dose reduction	10 (34.5 %)	1 (3.7 %)	0.010
At least one cycle delay	24 (82.8 %)	17 (63.0 %)	0.171
Second-line treatment with irinotecan	15 (51.7 %)	11 (40.7 %)	0.436
Third-line treatment	1 (3.4 %)	1 (3.7 %)	

^a^log-rank test

### Efficacy

#### Primary endpoint—overall survival

**Fig. 2:**
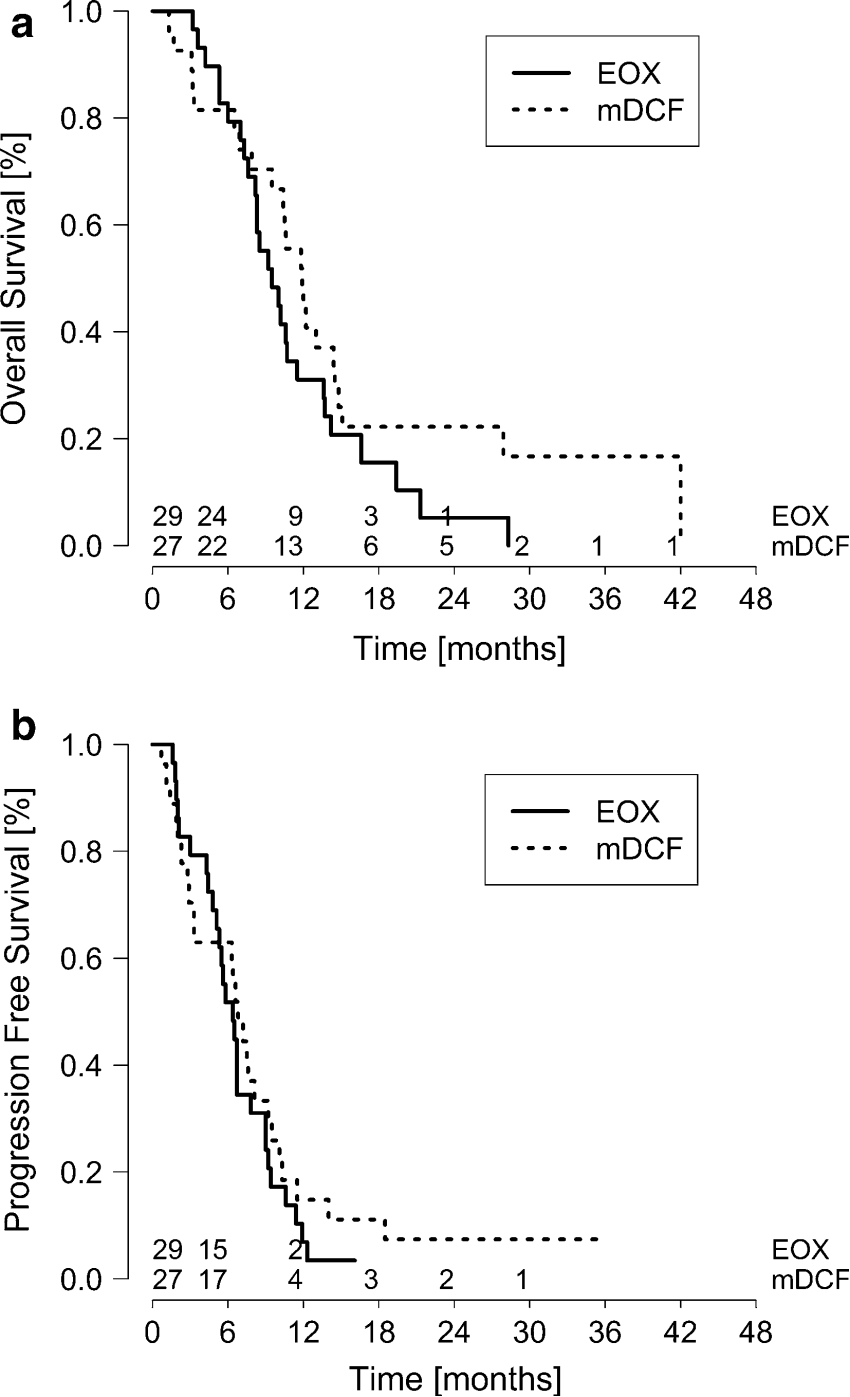
Kaplan–Meier estimates of **a** overall and **b** progression-free survival

During the follow-up (median follow-up of 34 months), 27 (93.1 %) patients on EOX and 23 patients (85.2 %) on mDCF had died. The median overall survival was 11.9 months in the mDCF compared to 9.5 months in the EOX arm (log-rank *p* = 0.135) (Fig. 2). The percentage of patients alive at 1 year was 44.4 % for mDCF and 31.0 % for EOX and at 2 years was 22.2 % for mDCF and 5.2 % for EOX (Table 2).

#### Secondary endpoint—progression-free survival

Progression-free survival did not differ significantly between study arms. The median progression-free survival was 6.8 months for mDCF and 6.4 months for EOX (log-rank *p* = 0.440).

### Safety

The safety analysis comprises all 29 patients from the EOX arm and 26 patients from the mDCF arm (Table 3). One patient from the mDCF arm experienced a rapid progression of the disease after having received the first cycle of chemotherapy. This patient did not appear in the department again and died soon, and the data on the toxicity of the administered first cycle of chemotherapy are unknown. Therefore, this patient is not included in the safety analysis.

**Table 3:** Most common treatment-related adverse events (safety population)

	EOX N = 29		mDCF N = 26	
	All grades	Grade 3 or 4	All grades	Grade 3 or 4
Anemia	13 (44.8 %)	2 (6.9 %)	16 (61.5 %)	2 (7.7 %)
Leukopenia	21 (72.4 %)	2 (6.9 %)	20 (76.9 %)	3 (11.5 %)
Neutropenia	25 (86.2 %)	21 (72.4 %)	22 (84.6 %)	13 (50.0 %)
Thrombocytopenia	6 (20.7 %)	0 (0.0 %)	5 (19.2 %)	0 (0.0 %)
Febrile neutropenia	0 (0.0 %)	0 (0.0 %)	1 (3.8 %)	1 (3.8 %)
Nausea	10 (34.5 %)	1 (3.5 %)	4 (15.4 %)	0 (0.0 %)
Vomiting	4 (13.8 %)	0(0.0 %)	3 (11.5 %)	0(0.0 %)
Diarrhea	5 (17.2 %)	1 (3.4 %)	5 (19.2 %)	1 (3.8 %)
Anorexia	8 (27.6 %)	2 (6.9 %)	7 (26.9 %)	2 (7.7 %)
Abdominal pain	4 (13.8 %)	0 (0.0 %)	2 (7.7 %)	0 (0.0 %)
Mucositis	2 (6.9 %)	0 (0.0 %)	4 (15.4 %)	0 (0.0 %)
Fatigue	9 (31.0 %)	2 (6.9 %)	6 (23.1 %)	1 (3.8 %)
Hand–foot syndrome	2 (6.9 %)	0 (0.0 %)	1 (3.8 %)	0 (0.0 %)
Thromboembolic events	4 (13.8 %)	1(3.4 %)	2 (7.7 %)	0 (0.0 %)
Peripheral neuropathy	2 (6.9 %)	0 (0.0 %)	4 (15.4 %)	0 (0.0 %)

There were no statistically significant differences between arms in toxicities of any grade or grade 3 or 4 (toxicity grade 3 or 4: EOX 79.3 % vs. mDCF 61.5 %, *p* = 0.234). However, neutropenia grade 3 or 4 was observed more frequently in the EOX arm (72.4 vs. 50.0 %; *p* = 0.153). Nausea of any grade was also observed more frequently in the EOX arm (34.5 vs. 15.4 %; *p* = 0.189). The rate of thromboembolic events was twice lower in the mDCF arm (7.7 vs. 13.8 %; *p* = 0.672). Interestingly, peripheral neuropathy was observed more frequently in the mDCF arm (15.4 vs. 6.9 %; *p* = 0.406).

The administration of supportive treatment during chemotherapy was similar in both arms, except for the granulocyte colony-stimulating factors (G-CSFs) which were used significantly more frequently in the mDCF arm (55.6 vs. 6.9 %; *p* < 0.001) (Table 4).

**Table 4:** Supportive treatment during chemotherapy

	EOX	mDCF	*p* value
G-CSFs	2 (6.9 %)	15 (55.6 %)	<0.001
Erythropoiesis-stimulating agents	3 (10.3 %)	4 (14.8 %)	0.700
Blood transfusion	3 (10.3 %)	3 (11.1 %)	1.000
Megestrol acetate	8 (27.6 %)	7 (25.9 %)	1.000

## Discussion

This randomized trial comparing two three-drug combination palliative chemotherapy regimens in advanced gastric and gastroesophageal junction HER2-negative adenocarcinoma showed that mDCF chemotherapy compared to EOX is associated with a 2.4-month longer overall survival with no increase in toxicity. However, this difference is not statistically significant.

The efficacy of the EOX regimen was first established in a randomized phase 3 trial REAL-2 [6], which evaluated substitution of 5-fluorouracil with capecitabine and cisplatin with oxaliplatin. The EOX regimen was associated with a statistically significant improvement in median overall survival compared to the original ECF regimen (11.2 vs. 9.9 months, *p* = 0.02) with no increase in toxicity. Therefore, the EOX has become a standard palliative chemotherapy regimen in many cancer centers. The V325 study [7] showed that the DCF combination (docetaxel/cisplatin/5-fluorouracil) is more effective than CF (cisplatin/5-fluorouracil) in terms of overall survival (9.2 vs. 8.6 months; log-rank *p* = 0.02), but it is associated with increased toxicity. Therefore, modifications of the DCF regimen were investigated with the aim of improving its tolerability. The mDCF regimen presented by Shah at al. [8] was shown to be at least as effective as and less toxic than the original DCF; the median overall survival of 15.1 months reached by patients treated with this combination was impressive. To our best knowledge, our study is the first head-to-head randomized comparison of the EOX and mDCF chemotherapy regimens.

The groups, although small, were well balanced in terms of the initial characteristics, except for more frequent malnutrition observed in the mDCF arm (18.5 vs. 3.5 %; *p* = 0.064) and more frequent liver metastases in this group of patients (48.1 vs. 17.2 %; *p* = 0.029). Andreyev et al. [9] showed that in gastric and gastroesophageal cancer patients, weight loss before the beginning of chemotherapy is an important prognostic factor and worse outcomes of these patients are attributable to higher toxicity of chemotherapy and consequently lower dose intensity. It has also been shown that the presence of liver metastases is associated with a worse overall survival [10, 11]. In this study, however, both malnutrition and liver metastases were observed more frequently in the mDCF arm, but still these patients had longer overall survival, which may suggest higher efficacy of this regimen.

Dose intensity in the EOX arm was lower compared to the mDCF arm. The mode of continuous capecitabine administration in the EOX regimen makes use of granulocyte colony-stimulating factors difficult in the case of hematological toxicity, especially neutropenia, which was the main reason for dose delays and/or dose reductions in the study. Indeed, dose reductions were more frequent in the EOX arm (34.5 vs. 3.7 %; *p* = 0.01) as well as dose delays (82.8 vs. 63.0 %; *p* = 0.171), but the second difference was not statistically significant. Significantly more patients in the mDFC arm were administered granulocyte colony-stimulating factors (55.6 vs. 6.9 %; *p* < 0.001), which allowed to maintain higher dose intensity in this group.

It is also possible that adherence to treatment in the EOX arm was lower. In theory, more frequent nausea observed in the EOX regimen (34.5 vs. 15.4 %; *p* = 0.189) might have negatively influenced the oral ambulatory administration of capecitabine. A reduced compliance with regard to the ambulatory administration of oral antineoplastic drugs is a well-known phenomenon. Although cancer patients prefer oral medications over intravenous therapy, about 20–30 % of them do not take pills regularly as recommended by their treating physician. Adverse effects of chemotherapy are among the main causes of reduced adherence to oral anticancer medications. It has been shown that adherence to oral medications correlates with treatment efficacy [12].

It has also been reported that first-line chemotherapy regimens containing docetaxel improve survival over chemotherapy combinations without a taxane. A meta-analysis of twelve randomized controlled trials including 1089 patients with palliatively resected, unresectable, recurrent or metastatic gastric carcinoma comparing first-line DCF chemotherapy with non-taxane-containing palliative regimens showed that DCF increases response rates and prolongs survival of patients with some increase in toxicity [13]. Therefore, the selection of a well-tolerated taxane-containing regimen in the first-line setting can potentially improve the outcomes.

There were no statistically significant differences in toxicity between the study arms; however, this lack of significance may result from the small number of patients. Nevertheless, grade 3 or 4 neutropenia was observed more frequently in the EOX arm (72.4 vs. 50.0 %; *p* = 0.153). The same refers to the rate of thromboembolic events which occurred two times more often in the EOX arm (13.8 vs. 7.7 %; *p* = 0.672). It has been proven that thromboembolic events are a negative prognostic factor shortening the survival of cancer patients [14, 15].

Our study has several limitations. First of all, this was a single-center study restricted to patients treated only in our department. Secondly, because of the limited access to CT, control imaging studies were performed every 8–12 weeks, in accordance with our routine clinical strategy. Therefore, there may be bias in the PFS assessment. And finally, the study was closed prematurely due to poor patients’ accrual and resulted in small sample size. It is therefore possible that if a small but true benefit existed in either group, this study may have been underpowered to detect it.

In conclusion, there is currently no one universal palliative chemotherapy regimen for the treatment of advanced gastric and gastroesophageal HER2-negative carcinoma. Our study did not show a statistically significant difference in median OS between compared arms. However, we believe that a 2.4-month longer median OS observed in the mDCF regimen is clinically important. Although mOS of our patients treated with the mDCF regimen was shorter than that reported by Shah et al. [8], it is still one of the longest observed in advanced gastric cancer. It is noteworthy that this gain in survival was reached without an increase in toxicity of the mDCF chemotherapy. Further randomized, large-scale trials are necessary to confirm our results.
